# HLA-A*0201-specific epitopes of Indian HIV-1C as candidates for vaccine design

**DOI:** 10.1186/1471-2334-12-S1-O15

**Published:** 2012-05-04

**Authors:** Jagadish Chandrabose Sundaramurthi, Luke Elizabeth Hanna

**Affiliations:** 1 Division of Biomedical Informatics, Department of Clinical Research, National Institute for Research in Tuberculosis (formerly Tuberculosis Research Centre) (ICMR), Chennai, 600031, Tamil Nadu, India; 2 Division of HIV/AIDS, Department of Clinical Research, National Institute for Research in Tuberculosis (formerly Tuberculosis Research Centre) (ICMR), Chennai, 600031, Tamil Nadu, India

## Background

HLA alleles are associated with differential outcomes of infections/diseases. We hypothesize that epitopes that interact with HLA alleles associated with resistance elicit a protective immune response in the host, and could therefore serve as good vaccine candidates. Among the HLA alleles reported to be associated with resistance to HIV infection/slow progression to AIDS, HLA-A*0201 occurs most frequently in the Indian population. We undertook this study to identify HIV epitopes specific to this HLA allele from HIV-1C.

## Methods

1769 sequences of all proteins of Indian HIV-1C were downloaded from the HIV sequence database and consensus sequence for each protein was built. Epitopes specific to HLA-A*0201, but not to any of the HLA alleles known to be associated with susceptibility to HIV/AIDS, were identified using ProPred1 and modeled on to HLA-A*0201.

## Results

Twenty six peptides specific to HLA-A*0201 were identified as better binders to the HLA molecule than a control peptide which is a known immunodominant HIV epitope. Eleven of these have also been reported by others as immunogenic. Ten of the 11 epitopes were found to be conserved in Indie-C1, an infectious Indian subtype C clone. Four epitopes were reported to elicit CD4+ as well as CD8+ specific responses. Fifteen epitopes identified in this study are novel.

## Conclusion

The short-listed epitopes could be tested for their potential as vaccine candidates for the Indian HIV epidemic. This approach has been extended to identify epitopes specific to other HLA alleles associated with resistance to HIV, and *in vitro* evaluation is being undertaken.

